# Case Report: Palopegteriparatide as a novel therapeutic option in pediatric autosomal dominant hypocalcemia type 1

**DOI:** 10.3389/fendo.2026.1837966

**Published:** 2026-06-17

**Authors:** Arkadiusz Zygmunt, Anna Fedorczak, Łukasz Krotowski, Anna Łupińska, Kinga Sałacińska, Agnieszka Gach, Michael Mannstadt, Renata Stawerska

**Affiliations:** 1 Department of Developmental Age and Adult Endocrinology, Medical University of Lodz, Lodz, Poland; 2 Department of Endocrinology and Metabolic Diseases, Polish Mother’s Memorial Hospital – Research Institute, Lodz, Poland; 3 Department of Genetics, Polish Mother’s Memorial Hospital – Research Institute, Lodz, Poland; 4 Endocrine Unit, Massachusetts General Hospital and Harvard Medical School, Boston, MA, United States

**Keywords:** ADH1, calcium-sensing receptor, hypoparathyroidism, palopegteriparatide, PTH analogs

## Abstract

**Introduction:**

Autosomal dominant hypocalcemia type 1 (ADH1) is a rare genetic disorder caused by gain-of-function variants in the *CASR* gene, leading to suppressed parathyroid hormone (PTH) secretion, hypocalcemia, hyperphosphatemia, hypomagnesemia and hypercalciuria. Evidence on the use of long-acting PTH analogs in pediatric ADH1 remains scarce. This report describes a boy with a *de novo* heterozygous activating *CASR* variant and highlights the therapeutic response to palopegteriparatide.

**Case presentation:**

A 16-year-old boy presented with hypocalcemia, recurrent tetany, seizures, hypercalciuria, nephrocalcinosis, basal ganglia calcifications, and early-onset cataracts. He had been diagnosed with congenital hypoparathyroidism at 6 weeks of age. A heterozygous *CASR* variant [c.2504C>A; p.(Ala835Asp)] causing ADH1 was identified. Despite long-term therapy with alfacalcidol, calcium, and magnesium supplements, symptoms persisted and biochemical control remained poor, with pronounced hyperphosphatemia and an elevated calcium–phosphate product.

**Intervention and outcomes:**

Owing to inadequate disease control on Standard-of-Care, off-label treatment with palopegteriparatide was initiated. The dose was adjusted to alleviate neuromuscular symptoms while reducing calcium-phosphate product. The patient subsequently reported complete resolution of tetany with improved biochemical stability, reduced phosphate levels, and a lower calcium–phosphate product. At the final maintenance dose, both alfacalcidol and calcium supplementation were successfully discontinued.

**Conclusion:**

This case demonstrates that palopegteriparatide may be a valuable therapeutic option in pediatric patients with ADH1 when conventional therapy fails to provide adequate control and while targeted therapies directed at the mutated CASR are not yet available. Carefully titrated PTH-based therapy can improve symptoms and mineral homeostasis, highlighting the need for further studies on its long-term safety and efficacy in children.

## 1 Introduction

Autosomal dominant hypocalcemia type 1 (ADH1 – OMIM 601198) is a rare genetic disorder caused by gain-of-function mutations in the *CASR* gene [1], leading to enhanced calcium-sensing receptor signaling and suppressed PTH secretion, hypocalcemia and hyperphosphatemia. Pronounced hypercalciuria is driven by two mechanisms, PTH deficiency and constitutive activation of the renal CASR and often leads to nephrocalcinosis and/or nephrolithiasis. Additionally, decreased magnesium levels may also be present (1).

The clinical manifestations of ADH1 vary in severity, largely depending on the extent of hypocalcemia and hyperphosphatemia. Common symptoms include neuromuscular irritability (such as paresthesias, muscle cramps, spasms, and tetany), seizures, arrhythmias, renal complications (including nephrocalcinosis, nephrolithiasis, and compromised kidney function), and the deposition of ectopic calcium in various organs (e.g., the brain) (2).

This case describes a 16-year-old boy with congenital hypoparathyroidism stemming from an activating variant of the calcium-sensing receptor *CASR*, NM_000388.4:c.2504C>A, p.Ala835Asp. Because of inadequate symptom control on conventional therapy, he was started on treatment with palopegteriparatide, a long-acting parathyroid hormone receptor type 1 (PTHR1) analog.

## 2 Case presentation

The patient was diagnosed with hypoparathyroidism at 6 weeks of age during hospitalization for a urinary tract infection. Total serum calcium measured twice was 5.5 mg/dL (range 7.8–11.3 mg/dL), phosphorus 11.2 mg/dL (range 4.8-8.2 mg/dL), magnesium 1.44 mg/dL (1.6–2.7 mg/dL), and parathyroid hormone (PTH) <3 pg/mL. The child did not exhibit clinical signs of hypocalcaemia, and calcium supplementation was initiated (2 x 115 mg Ca/day). The patient was subsequently rehospitalized approximately one month later at the Department of Pediatric Endocrinology, at which time the serum calcium was 7.4 mg/dL (8.4–11 mg/dL), PTH was markedly below the reference range, and 25-hydroxyvitamin D levels were undetectable. Since then, the patient has been under endocrinological and nephrological care. In his early years, he experienced recurrent urinary tract infections, for which he received prophylactic furazidinum until the age of two. Due to his hypoparathyroidism, he was treated with various doses of alfacalcidol or calcitriol, along with calcium and magnesium supplements. A low-phosphate diet was also recommended due to persistently elevated serum phosphate levels (6.6–9.7 mg/dl, range 4.5–6.5 mg/dl). He had previously received sevelamer periodically, but this pharmacotherapy was ineffective, caused significant abdominal pain and diarrhea and had to be discontinued.

Throughout the years, the boy frequently suffered from tingling, numbness, painful muscle cramps, and seizures occurring 1–2 times annually, necessitating hospitalizations. These symptoms of increased neuromuscular excitability were particularly exacerbated during infections, after physical exertion, and with exposure to high temperatures (e.g., in the summer). The clinical course was complicated by persistent hypercalciuria (urinary calcium excretion: 4.6-5.6 mg/kg/day; normal <4.0 mg/kg/day) and renal ultrasound at the age of 1.5 years revealed bilateral nephrocalcinosis and nephrolithiasis. Initiation of hydrochlorothiazide therapy resulted in a reduction in urinary calcium excretion; however, levels did not fully normalize. Imaging studies also revealed calcifications in the basal ganglia of the brain, and he was diagnosed with early-stage cataracts. In March 2010, at age almost 2 years, an allogeneic parathyroid cell transplant was performed; however, graft function was not achieved. Molecular testing in April 2015 at age 7 years identified the presence of a pathogenic variant in the *CASR* gene (see below). In December 2018, at the age of 10 years, the patient developed recurrent paroxysmal nocturnal episodes characterized by impaired awareness, focal motor phenomena, and post somnolence. These events were not associated with fever or acute tetany and occurred outside episodes of acute hypocalcemia (serum calcium levels around 7.0 mg/dL). The episodes were diagnosed as focal epilepsy by pediatric neurology specialists and treatment with oxcarbazepine was initiated. Both of his parents and his 3-year older brother are healthy. Although single measurements of serum total calcium showed slightly reduced levels in the mother (8.9 mg/dL) and father (8.5 mg/dL), and a normal level in the brother (9.4 mg/dL) (reference range 9.0-10.2 mg/dL), genetic testing excluded a diagnosis of ADH1. The boy attends a public school and excels in STEM subjects, particularly mathematics. He enjoys playing basketball and the piano.

### 2.1 Genetic testing

**Figure 1:**
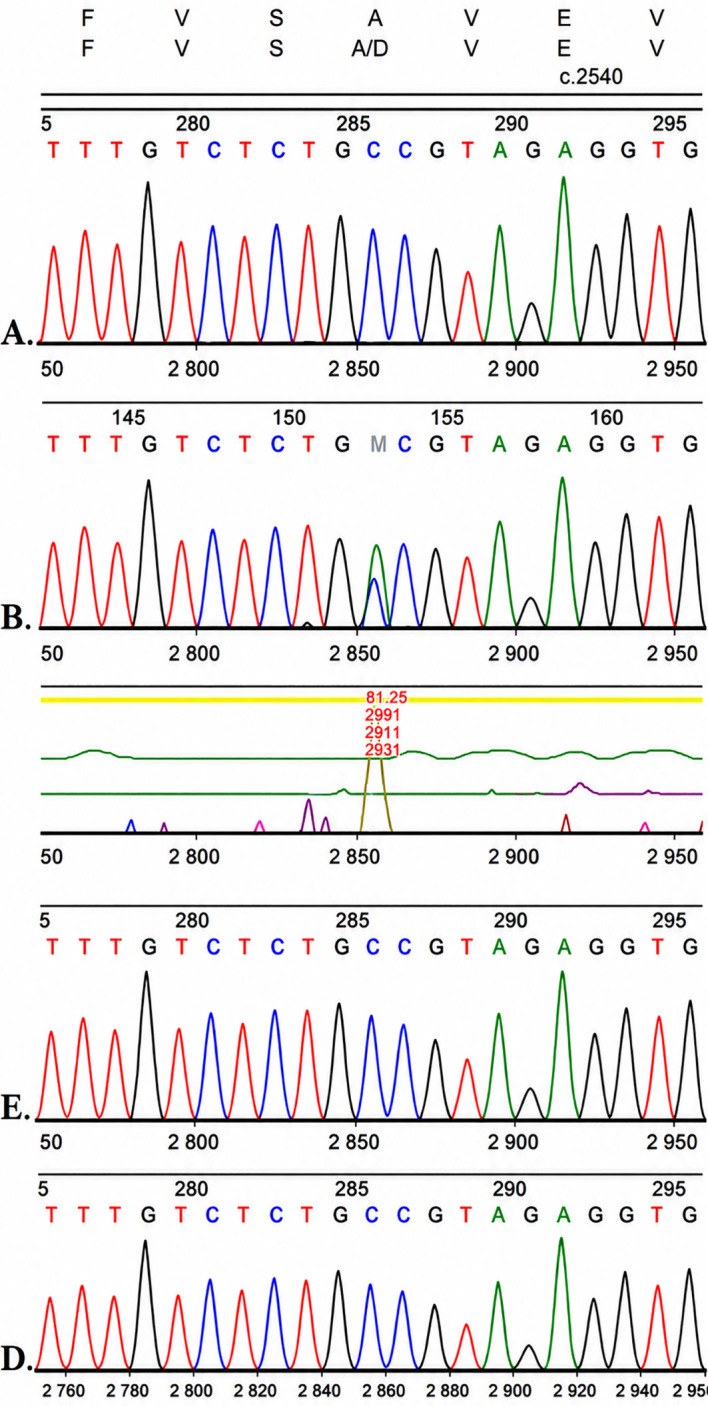
Sanger sequencing of the patient and his parents indicating *de novo* variant c.2504C>A (p.Ala835Asp) in the *CASR* gene. **(A)** Reference sequence. **(B)** The patient. **(C)** The patient's mother. **(D)** The patient’s father.

Molecular genetic analysis was performed at Oxford University Hospitals NHS Foundation Trust (Oxford, UK) using Sanger sequencing of the coding regions and exon–intron boundaries of the *CASR* and *GNA11* genes. A heterozygous pathogenic *CASR* variant, c.2504C>A (p.Ala835Asp), was identified in the proband. This missense variant has previously been reported in association with autosomal dominant hypocalcemia type 1 (ADH1) (Letz et al.). Subsequently, targeted Sanger sequencing was performed at the Polish Mother’s Memorial Hospital – Research Institute (PMMH-RI), and the variant was not identified in the patient’s parents, indicating a *de novo* origin of the c.2504C>A *CASR* gene variant (**Figure 1**).

### 2.2 Current clinical status

The patient has been under the care of the Department of Endocrinology and Metabolic Diseases at PMMH-RI in Lodz since 2020. Immediately prior to commencing palopegteriparatide, the patient was receiving alfacalcidol (1.25µg/day), CaCO3 (200 mg three times daily), hydrochlorothiazide (12.5 mg in the morning and 6.25 mg in the early afternoon), MgSO4 (260 mg Mg/day), and KCl (782 mg K/day). Interestingly, despite persistently low total serum calcium level (6.5-7.5 mg/dL, normal range 8.8-10.8 mg/dL), the patient remained relatively asymptomatic, with tetany occurring only sporadically (e.g., during viral infections or after significant physical activity). However, the patient presented with phosphate retention (TmP/GFR > 4.0 mmol/L), leading to hyperphosphatemia) (8.1-10.8 mg/dL; normal range 2.9-5.1 mg/dL) and an elevated calcium-phosphate product of 55–65 mg²/dL². The patient also exhibited increased fractional excretion of calcium (FECa>2.0%), hypercalciuria (393 mg/24 h), nephrocalcinosis, cataracts, and calcifications in the basal ganglia.

In September 2024 (at age 16 years 5 months), palopegteriparatide was initiated. As this medication is not registered for use in pediatric patients, informed consent for treatment was obtained from both parents and the patient. Furthermore, the treatment request was approved by the Bioethics Committee of the PMMH-RI.

## 3 Intervention and outcomes

Treatment with palopegteriparatide began in mid-September 2024 (at age 16 years 5 months, weight 61 kg, height 175 cm) at an initial dose of 9 µg/day (the recommended starting dose in adults is 18 mcg/day). The medication dosage was carefully adjusted to prevent symptoms of increased neuromuscular excitability while simultaneously aiming for the lowest possible calcium-phosphate product. The patient’s measurements, laboratory parameters and BMD results, both pre-treatment and during palopegteriparatide administration, are presented in **Table 1**. Although the initial dose of the drug lowered serum phosphate levels, they remained above the normal range. At the same time, serum calcium increased, consequently elevating the calcium–phosphate product. Therefore, careful adjustment of the palopegteriparatide dose was essential. Ultimately, the palopegteriparatide dose was reduced to 7.5 µg/day alfacalcidol and calcium supplements were discontinued and hydrochlorothiazide was lowered to 6.25 mg/day. Supplementation with magnesium (260 mg Mg/day) and potassium (391 mg K/day) was maintained. **Table 2** illustrates the medication dosage adjustments based on biochemical analyses.

**Table 1:** Laboratory, anthropometric, and bone mineral density parameters before and during palopegteriparatide treatment.

Variable			Time period			
Anthropometry			Before	3 months	6 months	12 months
Age [years, month]			16y 5m	16y 8m	16y 11m	17y 5m
Height [cm]			175	N/A	176	176
Body mass [kg]			61	N/A	65	65
Fat [%]			19.1	N/A	18.3	20.0
Fat [g]			11965	N/A	11940	12026
Lean mass [g]			47556	N/A	50187	48003
Laboratory Data	Unit	Range				
Calcium	[mg/dL]	8.8 - 10.8	6.52	7.80	7.52	7.56
Calcium (ionized)	[mmol/L]	1.2 - 1.32	0.82	0.97	0.98	0.96
Albumin	[g/dL]	4.0 - 5.2	4.7	5.1	4.8	5.2
Phosphate	[mg/dL]	2.88 - 5.05	8.71	6.40	6.67	6.94
Creatinine	[mg/dL]	0.72 - 1.18	0.89	0.80	0.82	0.87
Magnesium	[mg/dL]	1.99 - 2.71	1.59	N/A	1.8	1.8
Ca x P	[mg^2^/dL^2^]	< 55	56.8	49.92	50.15	52.5
PTH	[pg/mL]	14.9 - 56.99	2.9	3.0	2.9	<5.5
FGF-23	[pg/mL]	23.2 - 95.4	75.0	59.1	46.8	N/A
25(OH)D	[ng/mL]	30.4 - 49.7	27.6	22.5	33.3	30.2
1,25(OH)_2_D	[pg/mL]	19.9 - 79.3	23.9	40.4	37.6	N/A
Osteocalcin	[ng/mL]	N/A	77.0	95.8	91.6	87.9
*β*-CrossLaps	[pg/mL]	N/A	1902	2034	1807	1619
Alkaline phosphatase	[U/L]	90 - 377	N/A	144	136	123
FE Ca	[%]	< 1	2.18	0.52	1.22	0.7
24-hour urinary calcium	[mg/24h]	40-220	393	266**	173***	85****
24-hour urinary magnesium	[mg/24h]	73-122	N/A	202.6**	164.2***	123.4****
TRP		0.78-0.91	0.99	0.99	0.98	1
TmP/GFR (TRP ≥ 0.86)	[mmol/L]	0.84 - 1.23	4.08	2.97	2.91	2.23
TSH	[mIU/L]	0.51 - 4.3	2.19	0.97	1.22	1.29
FT_4_	[pg/mL]	0.98 - 1.63	1.13	1.48	1.36	1.29
FT_3_	[ng/mL]	2.56 - 5.01	3.48	3.62	3.72	4.08
Na	[mmol/L]	134 - 143	144	141	140	140
K	[mmol/L]	3.5 - 5.1	4.4	4.1	4.0	3.9
Cl	[mmol/L]	97 - 110	103	103	104	N/A
Alanine aminotransferase	[U/L]	10 - 24	11	10	11	N/A
Aspartate aminotransferase	[U/L]	16 - 37	28	25	29	N/A
Bilirubin (total)	[mg/dL]	0.2 - 0.9	1.2	1.1	1.3	N/A
Urea nitrogen	[mg/dL]	17 - 43	27.1	38.6	34.9	N/A
BMD [g/cm^2^ ]*						
Spine		L1	1.319	N/A	1.361	1.308
Spine		L2	1.442	N/A	1.479	1.459
Spine		L3	1.447	N/A	1.482	1.458
Spine		L4	1.356	N/A	1.371	1.402
Spine		L1-L4	1.392	N/A	1.423	1.410
Spine		L2-L4	1.412	N/A	1.440	1.438
Hip		femur	1.212	N/A	1.159	1.183
Hip		total	1.347	N/A	1.275	1.262
Forearm		⅓ forearm	0.736	N/A	0.757	0.767
Forearm		total	0.597	N/A	0.612	0.599

Ca × P, calcium–phosphate product; PTH, parathyroid hormone; FGF-23, fibroblast growth factor 23; 25(OH)D, 25-hydroxyvitamin D; 1,25(OH)_2_D, 1,25-dihydroxyvitamin D; β-CrossLaps (CTX), beta-C-terminal telopeptide; FE Ca, fractional excretion of calcium; TRP, tubular reabsorption of phosphate; TmP/GFR, tubular maximum for phosphate reabsorption per GFR; TSH, thyroid-stimulating hormone; FT4, free thyroxine; FT3, free triiodothyronine; LSC, Least Significant Change.

*LSC (Spine, children) – 0.017 g/cm2 at 95% confidence level, LSC (Spine, adults) – 0.058 g/cm2 at 95% confidence level, LSC (Femur, adults) – 0.023 g/cm2 at 95% confidence level.

** after 4 months; *** after 6 months, ****after 16 months, performed separately from other urine analyses.

**Table 2:** Medication dosage adjustments based on biochemical analyses.

Date	Treatment					Serum			Urine		
	Palopegteriparatide	1(OH)D	CaCO_3_	Hydrochlorothiazid	KCl tabl/d	Calcium	Phosphate	Ca × P	FE Ca	TRP	TmP/GFR
	(µg/d)	(µg/d)	(mg/d)	(mg/d)	1 tabl = 391 mg K^+^	(mg/dL)	(mg/dL)	(mg²/dL²)	(%)		(mmol/L)
19.09.2024	9.0	1.25	1500	12.5–6.25	2	6.5	8,7	56.8	2.2	0.99	4.1
23.09.2024	9.0	1	1500	12.5–6.25	2	8.8	6,0	52.8			
24.09.2024	9.0	0	0	12.5–6.25	2						
26.09.2024	9.0	0.5	0	12.5–6.25	2	8.1	5.5	44.2	1.0	1.02	2.5
27.09.2024	9.0	0	0	12.5–6.25	2						
29.09.2024	9.0	0	0	0	2						
3.10.2024	9.0	0	0	0	2	7.7	5.8	44.7	0.6	0.98	2.6
15.10.2024	6.0	0	0	0	2	8.7	6.5	56.5	1.5	0.93	2.3
23.10.2024	6.0	0	0	0	2	7.8	6.4	49.9	0.6	0.98	2.8
30.10.2024	6.0	0	0	0	2	7.6	6.2	47.1	1.3	0.97	2.6
8.11.2024	6.0	0	0	0	2	6.9	7.2	49.5	1.2	0.98	3.2
9.11.2024	6.0	0	0	12.5–6.25	2						
20.11.2024	6.0	0	0	12.5–6.25	2	6.7	7.7	51.6	0.5	0.97	3.2
27.11.2024	6.0	1	0	12.5–6.25	2	7.6	7.4	56.2	0.5	0.96	2.7
29.11.2024	6.0	0	0	12.5–6.25	2						
1.12.2024	9.0	0.25	0	12.5–6.25	2	7.6	6.7	50.9	0.7	0.96	2.7
4.12.2024	9.0	0.25	0	12.5–6.25	2	7.8	6.4	49.9	0.5	0.96	3.0
13.12.2024	9.0	0.25	0	6.25–6.25	2	9.2	6.4	58.9	2.1	0.99	3.0
31.12.2024	9.0	0.25	0	6.25–6.25	2	7.7	6.4	49.6	1.0	0.96	2.9
10.01.2025	6.0	0	0	6.25–6.25	2	7.9	7.0	55.3	0.5	0.98	3.4
31.01.2025	6.0	0	0	6.25	2						
7.02.2025	6.0	0	0	6.25	2	6.6	8.2	54.3	0.5	0.99	3.8
24.02.2025	7.5(5x) 6.0 (2x)/week	0	0	6.25	2	6.9	7.5	51.8	0.6	0.99	3.4
16.03.2025	7.5	0	0	6.25	2						
8.04.2025	7.5	0	0	6.25	1	7.5	6.7	50.2	1.2	0.98	2.9
9.05.2025	7.5	0	0	6.25	1	7.2	7.6	54.7	1.1	0.97	3.2
13.06.2025	7.5	0	0	6.25	1	6.9	7.9	54.1	0.5	0.99	3.5
18.07.2025	7.5(5x) 6.0 (2x)/week	0	0	6.25	1	8.2	6.8	55.7	2.2	0.99	3.1
22.08.2025	7.5	0	0	6.25	1	8.5	6.6	56.0	2.2	0.98	2.9
23.08.2025	7.5(5x) 6.0 (2x)/week	0	0	6.25	1						
26.09.2025	7.5(5x) 6.0 (2x)/week	0	0	6.25	1	7.6	6.9	52.5	0.7	1.00	3.3
29.10.2025	7.5(4x) 6.0 (3x)/week	0	0	6.25	1	7.5	6.9	52.2	1.2	1.00	3.3

CaCO_3_, calcium carbonate; KCl, potassium chloride; Ca×P, calcium–phosphate product; FE Ca, fractional excretion of calcium (%); TRP, tubular reabsorption of phosphate; TmP/GFR, tubular maximum phosphate reabsorption corrected for glomerular filtration rate; d, day.

After 12 months of treatment, the patient was asymptomatic, with no episodes of tetany. The calcium level increased to 7.52 mg/dL (peak – 9.2 mg/dL), phosphate levels decreased to 6.94 mg/dL (nadir – 5.46 mg/dL), and the calcium-phosphate product dropped from 56.8 mg²/dL² (range 55–68) to 52.2 mg²/dL² (range 44.2–58.9). Bone turnover markers and BMD also were monitored during follow-up, with no evidence of excessive skeletal effects.

## 4 Discussion

The CaSR receptor, which forms a dimer, plays a key role in calcium homeostasis (3). This G-protein coupled receptor comprises 1078 amino acids and consists of a large extracellular domain (ECD), a seven transmembrane domain (7TMD) and an intracellular domain (ICD) (4, 5). Its highest expression is in the parathyroids and kidneys, where it regulates PTH secretion and renal calcium reabsorption in response to the extracellular calcium concentration, respectively. Although *CASR* is also expressed in other tissues such as pancreas, bone, brain, skin, lens epithelium, thyroid, intestine, lung and heart, its function in these organs remains largely unknown (3, 6).

Pathogenic inactivating (loss-of-function) variants in *CASR* gene cause familial hypocalciuric hypercalcaemia (FHH) type 1, neonatal severe primary hyperparathyroidism (NSPHT), whereas, activating (gain-of-function) variants lead to ADH1 and ADH1 with Bartter syndrome (BS). Accordingly, FHH and NSPHT are associated with hypercalcemia, while ADH1 and ADH1 with BS with hypocalcemia (4, 5, 7).

To date, the Leiden Open Variation Database (LOVD) lists over 430 *CASR* variants, including approximately 105 associated with ADH1, 155 with FHH, 31 with NSPHT and 14 with mixed FHH and NSPHT phenotypes (8). Recently, CASRdb, a publicly accessible comprehensive database compiling disease-associated CASR variants from the literature and genomic repositories, has been developed (9). According to CASRdb, 498 disease-associated CASR variants have been reported to date, including 121 (24.3%) linked to ADH1 and 377 (75.7%) to FHH1. Both inactivating and activating variants are dispersed however there is a clustering in exons 3, 4, and 7 (“hot spots”), for both types of pathogenic variants (5). Estimates for the prevalence of ADH1 range from 0.3/100,000 to 3.9/100,000 (1, 10, 11).

### 4.1 Molecular and functional aspects

Under physiological conditions, increased extracellular calcium activates CaSR leading to suppression of PTH secretion and reduced renal tubular calcium reabsorption (4). Activating *CASR* mutations increase the receptor’s sensitivity to extracellular calcium, leading to inappropriately low PTH secretion and excessive urinary calcium loss—resulting in hypocalcemia, hyperphosphatemia, and hypercalciuria (3). Hypercalciuria in ADH1 is driven by a dual mechanism—PTH deficiency and constitutive activation of the renal CaSR—leading to a high prevalence of nephrocalcinosis and nephrolithiasis and renal impairment, complications shown to worsen under conventional standard-of-care therapy (1). In addition, hypomagnesemia, frequently observed in ADH1, is primarily related to renal magnesium wasting due to CaSR overactivation in the thick ascending limb (12). Importantly, low magnesium levels may further suppress PTH secretion, thereby exacerbating hypocalcemia and contributing to the severity of the biochemical phenotype (13).

In our patient, a heterozygous c.2504C>A variant in exon 7 of CASR gene was identified, consistent with ADH1. According to The American College of Medical Genetics and Genomics (ACMG) criteria, the c.2504C>A variant is classified as pathogenic. It was previously reported in a patient with hypocalcemia (3), submitted to ClinVar (ID: 3263506), is absent from population databases and listed in CASRdb as an activating variant associated with ADH1 (9). The variant causes a missense substitution for alanine at position p.835, which is highly conserved and located within the functionally important transmembrane domain 7, specifically extracellular loop 3 of the 7TMD, a known hotspot for activating ADH1 mutations (4). Variants in this region are thought to stabilize the CaSR in its active conformation (7). Functional studies of substitutions of the same amino acid alanine in position 835 with threonine or aspartic acid (p.Ala835Thr or p.Ala835Asp) have demonstrated activation of cytosolic calcium signaling at subphysiological extracellular calcium concentrations (3, 7). In silico prediction tools unanimously support a deleterious effect on the gene (https://franklin.genoox.com/clinical-db/home). Recently, Harada and Namba reported additional cases involving the same p.Ala835Asp CASR variant in two sisters presenting with early-onset hypocalcemia, seizures, and nephrocalcinosis despite conventional therapy. Notably, their father, who carried the same variant, remained asymptomatic, highlighting the variable expressivity associated with ADH1. Importantly, their report further underscores the difficulty of achieving stable biochemical control with conventional treatment, consistent with the clinical course observed in our patient (14).

### 4.2 Differential diagnosis

The differential diagnosis of ADH1 includes several conditions affecting PTH secretion and calcium homeostasis. Other forms of hypoparathyroidism, particularly postoperative hypoparathyroidism, predominate in adults, whereas genetic etiologies are more common in children. Nevertheless, in all cases of hypocalcemia without a history of cervical surgery, genetic evaluation should be offered. Among genetic causes, autosomal dominant hypocalcemia type 2 (ADH2), caused by activating mutations in GNA11, represents the principal alternative diagnosis to ADH1, as it presents with a similar biochemical profile, but typically lacks hypercalciuria and hypomagnesemia (15, 16). Other inherited forms of hypoparathyroidism include mutations in genes such as GCM2, PTH, as well as syndromic entities such as DiGeorge (22q11.2 deletion) and HDR (hypoparathyroidism–deafness–renal dysplasia) syndromes (13, 17). Furthermore, autoimmune and infiltrative diseases leading to hypoparathyroidism as well as hypomagnesemia leading to a reversible, functional hypoparathyroidism, should also be considered (18). Distinguishing ADH1 from these other forms relies on positive family history, the coexistence of hypercalciuria, hyperphosphatemia, low PTH, hypomagnesemia, and genetic confirmation of an activating *CASR* variant.

### 4.3 Therapeutic implications

The current standard of care for patients with ADH1 is based on active vitamin D analogues (alfacalcidol or calcitriol) combined with oral calcium supplementation, as in other forms of hypoparathyroidism. However, it is important to emphasize certain therapeutic distinctions specific to this condition. Gain-of-function mutations in *CASR* gene increase renal calcium excretion more than in other forms of hypoparathyroidism; attempts to increase serum calcium will worsen hypercalciuria and may increase the CaxP product leading to nephrocalcinosis, nephrolithiasis, renal impairment and calcifications of other tissues (2, 19). Therefore, the target calcium concentration should remain below the lower limit of normal to minimize hypercalciuria, while maintaining the patient with minimal neuromuscular symptoms. Vitamin D analogs and calcium should be used cautiously under close biochemical monitoring, balancing symptom relief with renal safety (2). Thiazide diuretics can also be considered in ADH1, given their ability to mitigate hypercalciuria through enhanced calcium reabsorption in the distal tubules (20).

Understanding the pathogenesis of the disease is also crucial in managing emergency situations in patients with ADH type 1. As a rule, intravenous calcium salts are administered to terminate an acute tetany episode rather than to correct chronic calcium deficiency. However, it is difficult to expect that a physician providing emergency care—without access to patient’s medical history—would refrain from continuing the administration of intravenous calcium once the acute situation has been treated, but when the serum calcium levels are still markedly below the normal range; even in an asymptomatic patient (as in our case, with levels of 6.5–7.0 mg/dL). The situation is further complicated by the fact that each patient may have a different CaSR “set point,” for symptoms. Therefore, patients should be informed about their condition and provided with medical alert documentation, including a written explanation to be presented in the event of emergency hospitalization.

The ideal therapeutic strategy in ADH1 should counteract CaSR overactivation. Calcilytics, such as encaleret, function as negative allosteric CaSR modulators and represent a rational, disease-specific approach (21, 22). Data from the phase 2 clinical trial and preliminary results from the phase 3 CALIBRATE trial (23, 24) demonstrate normalization of serum calcium, phosphate and magnesium levels as well as 24-hour urine calcium, in patients aged ≥16 years with ADH1. However, this drug has not yet been approved and a pediatric trial is about to start.

In our patient, an alternative pharmacotherapy option was the administration of palopegteriparatide, a novel long-acting prodrug designed to release PTH(1-34). It is administered once daily by subcutaneous injection and is designed to provide stable, physiological hormone replacement in hypoparathyroidism. In the phase 3 PaTHway clinical trial, which included patients ≥18 years, palopegteriparatide demonstrated efficacy, safety, and tolerability in adults with hypoparathyroidism, and it has been approved for use in adults in the EU, UK, USA, and Australia (25–27). However, at the time of this writing, there is no published data on its use in children. To our knowledge, no pediatric cases of ADH1 treated with palopegteriparatide have been reported to date.

In our patient, this therapeutic approach aimed to improve hypocalcemia, alleviate neuromuscular symptoms, reduce hyperphosphatemia, and achieve normal urinary calcium levels while lowering the calcium–phosphate product to prevent ectopic calcifications. Careful dose titration was essential, as excessive exposure to PTH analog could induce hypercalcemia, exacerbate hypercalciuria leading to an increased risk of nephrocalcinosis. Although the recommended starting dose of palopegteriparatide in adults is 18 mcg/day, the decision was made to initiate treatment at a lower dose — 9 mcg/day for several clinical and practical reasons. It was assumed that the dose would be sufficiently low, and its effect modest enough for alfacalcidol and/or calcium supplementation to remain necessary, permitting gradual up-titration of the drug while progressively reducing—and ultimately discontinuing—calcium and active vitamin D. Additionally, given the available dose ranges and the reimbursement rules of the Polish National Health Fund—together with the patient’s body weight—it was also more practical to begin with a lower dose.

In the PaTHway trial (28), participants included both individuals with iatrogenic hypoparathyroidism (n=70) and those with other etiologies (n=12). Among the latter, there were 7 patients with idiopathic hypoparathyroidism, 2 with autoimmune polyendocrine syndrome type 1 (APS-1), 1 with ADH, 1 with HDR, and 1 with DiGeorge syndrome. The authors did not report differences in dosing requirements; however, this subgroup was small compared with the subgroup with postsurgical disease and was highly heterogeneous in terms of etiopathogenesis. A similar concern applies to the relationship between drug dose and body weight. Is it reasonable to assume that a patient weighing 100 kg requires the same dose as someone weighing 50 kg? The PaTHway trial does not provide an answer to this question (28), yet we, as endocrinologists, are well aware that body weight has a significant impact on hormone dosing as exemplified by levothyroxine. We therefore conclude that the dose of palopegteriparatide required to achieve biochemical control of hypoparathyroidism is highly variable and must be individualized; with consideration of lower starting doses in pediatric patients, whose body weight is typically lower than that of adults. Currently, no evidence-based weight-adjusted dosing recommendations for palopegteriparatide are available, particularly in pediatric populations.

Ultimately, the initial dose of palopegteriparatide (9.0 mcg/day) was reduced to 7.5 mcg/day, which resulted in satisfactory control of clinical symptoms. This dose adjustment allowed discontinuation of alfacalcidol and calcium supplementation, as well as led to a sustained reduction of the calcium–phosphate product (although not its normalization, due to persistently elevated phosphate levels).

Overall, the patient was asyptomatic, with no recurrence of tetany during the twelve months of the therapy. While the long-term safety of sustained PTHR1 analog administration and standardized monitoring strategies remains to be established, this treatment may represent a valuable option in selected children with hypoparathyroidism, including genetic forms, which are not controlled with standard of care. However, as a single case report, our findings are inherently limited in generalizability and should be interpreted with caution.

## 5 Conclusions

This case illustrates the complex pathophysiology and management challenges of patients with ADH1 which is due to activating variants in the *CASR*. Palopegteriparatide, a long-acting PTH(1-34), provided effective symptom control and improvement in calcium-phosphate imbalance compared to standard of care in our patient. Although long-term outcomes and pediatric experience remain limited, PTH-based therapy may be considered in children with hypoparathyroidism with severe biochemical disturbances refractory to conventional treatment and while targeted therapies directed at the mutated *CASR* are not yet available.

## Data Availability

The data presented in the study is deposited in the ClinVar (NIH) repository, submission number SCV007592733.

## In memoriam

We would like to acknowledge Professor Andrzej Lewiński, who was the first member of our team to initiate the patient’s treatment in 2010. Unfortunately, due to his sudden death in October 2024 he was unable to participate in the preparation of this manuscript; however, he remained an integral member of the research team.
